# HDAC2‐Mediated SMAD7 Stabilisation Activates Wnt/β‐Catenin Signalling to Drive DNA Damage Repair and Cisplatin Resistance in Ovarian Cancer

**DOI:** 10.1111/jcmm.71300

**Published:** 2026-08-03

**Authors:** Yingying He, Meiling Wu, Xiaomin Xu, Kang Zheng, Kening Zhou

**Affiliations:** ^1^ Department of Pathology Quzhou People's Hospital, The Quzhou Affiliated Hospital, Wenzhou Medical University Quzhou Zhejiang China; ^2^ Department of Gynaecology Quzhou People's Hospital, The Quzhou Affiliated Hospital, Wenzhou Medical University Quzhou Zhejiang China; ^3^ Department of Traditional Chinese Medicine Quzhou People's Hospital, The Quzhou Affiliated Hospital, Wenzhou Medical University Quzhou Zhejiang China

**Keywords:** deacetylation, DNA damage repair, HDAC2, ovarian cancer, SMAD7

## Abstract

To investigate the role of histone deacetylase 2 (HDAC2) in cisplatin resistance in ovarian cancer (OC). Cisplatin‐resistant OC cell lines were employed to construct HDAC2 overexpression and knockdown models, and their effects on cell proliferation and apoptosis were examined. Chromatin immunoprecipitation, immunoprecipitation, and dual‐luciferase reporter assays were performed to investigate the regulation of SMAD7 protein stability and promoter activity by HDAC2. Expression of DNA damage repair‐related genes was detected by qRT‐PCR. A xenograft mouse model was established for in vivo validation. HDAC2 was highly expressed in cisplatin‐resistant OC cells. Overexpression of HDAC2 enhanced drug resistance and inhibited apoptosis and DNA damage, whereas knockdown of HDAC2 exhibited the opposite effects. Mechanistically, HDAC2 directly deacetylated the SMAD7 protein to prevent its degradation rather than suppressing its transcription via H3K27 deacetylation. The HDAC2/SMAD7 axis promoted drug resistance by activating the Wnt/β‐catenin signalling pathway and modulating DNA damage repair‐related genes. In vivo experiments confirmed that HDAC2 knockdown significantly inhibited tumour growth and enhanced the sensitivity. HDAC2 enhances cisplatin resistance in OC by deacetylating and stabilising SMAD7 protein, thereby activating the Wnt/β‐catenin signalling pathway and promoting DNA damage repair.

## 1 Introduction

Ovarian cancer (OC) is one of the most lethal malignancies of the female reproductive system, with a poor prognosis primarily attributed to difficulties in early diagnosis and the development of chemotherapy resistance, particularly platinum resistance [1]. As a cornerstone drug for advanced OC, carboplatin triggers tumour cell apoptosis through DNA damage, yet the clinical onset of resistance is frequently driven by the activation of robust DNA damage repair pathways [2]. However, tumour cells can develop resistance through multiple mechanisms, including drug efflux, DNA damage repair, transcriptional and post‐translational regulation, and apoptosis evasion [3]. Among these, alterations in histone modifications play a critical role in chemotherapy resistance [4].

Histone deacetylases (HDACs) are a family of enzymes that catalyse the deacetylation of histone lysine residues, and their aberrant activity is closely associated with malignant tumour progression [5]. HDAC2, an important member of class I HDACs, is abnormally expressed in various cancers and is closely related to cell proliferation, apoptosis evasion, and chemotherapy resistance [6]. Studies have shown that high expression of HDAC2 may influence the DNA damage response and apoptotic threshold in tumour cells by regulating the expression of specific genes [7, 8]. However, the precise role of HDAC2 in cisplatin resistance in OC and its downstream regulatory network remain incompletely understood.

In exploring the key downstream effector molecules of HDAC2, SMAD family member 7 (SMAD7) has attracted our attention. SMAD7 is not only a critical negative regulator of the transforming growth factor‐β (TGF‐β) signalling pathway but also participates in DNA damage response, cell cycle regulation, and apoptosis control [9]. Moreover, gene expression can be modulated by epigenetic modifications, such as histone acetylation, which often influence promoter accessibility [10]. For instance, H3K27 acetylation (H3K27ac), as a hallmark of active transcriptional regions, may directly regulate SMAD7 transcription [11]. However, whether HDAC2 influences SMAD7 expression by modulating the histone acetylation status of its promoter region, thereby mediating cisplatin resistance, remains unclear.

Therefore, this study systematically investigated the function of HDAC2 in cisplatin resistance in OC and its post‐translational regulatory mechanisms. We confirmed in cell models that high HDAC2 expression promotes cisplatin resistance and inhibits cell apoptosis and DNA damage. Through functional experiments, we further elucidated that HDAC2 acts as a critical stabiliser of the SMAD7 protein. The HDAC2‐mediated deacetylation of SMAD7 prevents its hyperacetylation‐induced degradation, thereby sustaining the downstream Wnt/β‐catenin signalling and DNA damage repair. Additionally, animal experiments validated the potential of targeting HDAC2 to reverse cisplatin resistance. This study provides a novel perspective for elucidating the protein stability‐based regulatory mechanisms of cisplatin resistance in OC and suggests that the HDAC2/SMAD7 axis may serve as an effective target for overcoming chemotherapy resistance.

## 2 Materials and Methods

### 2.1 Cell Culture

Human OC cell lines (A2780, SKOV3, purchased from the American Type Culture Collection; Manassas, VA, USA) were cultured in RPMI‐1640 medium (Gibco, CA, USA) supplemented with 10% fetal bovine serum (FBS) and 1% penicillin/streptomycin in a humidified incubator at 37°C with 5% CO_2_. The cisplatin‐resistant cell lines (A2780/DDP, SKOV3/DDP) were obtained from the Cell Storage Centre of Wuhan University (China) and cultured under the same conditions.

### 2.2 Cell Transfection

For gene overexpression or knockdown, cells were seeded in 6‐well plates or culture dishes 24 h before transfection, and transfection was performed when the cells reached 60%–70% confluence. The HDAC2 overexpression plasmid (oeHDAC2) and its empty vector control (Vector), shRNA plasmid vectors for the HDAC2 and SMAD7 genes (sh‐HDAC2, sh‐SMAD7), and the negative control shRNA (sh‐NC) were transfected using Lipofectamine 3000 (Invitrogen) according to the manufacturer's instructions. All plasmids were purchased from Shanghai GenePharma (China).

### 2.3 Quantitative Real‐Time PCR (qRT‐PCR)

Total RNA was extracted from cells using TRIzol reagent (Invitrogen, CA, USA). RNA was reverse transcribed into cDNA using the PrimeScript RT reagent kit (Takara, Tokyo, Japan). Subsequently, qPCR reactions were performed using the SYBR Green Premix Pro Taq HS qPCR Kit (Accurate Biology, Hunan, China). Gene expression levels were calculated using the 2^−ΔΔCt^ method and normalised to GAPDH as the internal reference gene. Primer sequences are listed in Table 1.

**TABLE 1:** The primer sequences.

Name	Primer	Sequences (5′‐3′)
HDAC2	Forward	ATGGGTGGGAGGTCAAATGT
	Reverse	AGTTTAGGCGCCACTGTTTG
SMAD7	Forward	GGGGAGGGGAGGAATGAAAA
	Reverse	CCCACAGATCCAACTCCACT
RAD51	Forward	TCCCCAGAACAGCAGCATTA
	Reverse	GGTTGCTAGCCCGAGTATCT
ERCC1	Forward	GATCTGTCGCGTGGTCTTTC
	Reverse	GATACAGAGATCAGGGCCCC
UHRF1	Forward	TTGGGGCTGGATCATTGTCT
	Reverse	AAGGTGTCTGGGGATGTCTG
EXO1	Forward	TGATGACGGTGATGGCGATA
	Reverse	TGTGCACCCCGATATACTCC
MCM3	Forward	TCCTGGGGCTTTTCTGGTAG
	Reverse	TGGCCAGTCGAATCAGAGTT
GAPDH	Forward	CGGAGTCAACGGATTTGGTCGTAT
	Reverse	AGCCTTCTCCATGGTGGTGAAGAC

### 2.4 Western Blotting

Total protein was extracted from cells using RIPA lysis buffer. Equal amounts of protein samples were separated by SDS‐PAGE gel electrophoresis and transferred to PVDF membranes. Membranes were blocked with 5% non‐fat milk for 1 h, followed by incubation with specific primary antibodies overnight at 4°C. Primary antibodies used included the following: HDAC2 (1:1000, ab219053, Abcam), SMAD7 (1:1000, ab216428, Abcam), cleaved PARP1 (1:1000, ab32064, Abcam), cleaved caspase‐3 (1:500, ab32042, Abcam), Ɣ‐H2AX (1:1000, ab81299, Abcam), β‐catenin (1:5000, ab32572, Abcam), histone H3 (as a nuclear protein loading control; 1:1000, ab1791, Abcam) and GAPDH (as a total protein loading control; 1:10000, ab128915, Abcam). The following day, membranes were incubated with the corresponding horseradish peroxidase‐conjugated secondary antibody (1:2000, ab181658, Abcam) for 1 h at room temperature. Protein bands were developed using an ECL chemiluminescence kit (Millipore, MA, USA) and detected by a chemiluminescence imaging system (Bio‐Rad).

### 2.5 Cell Viability and Proliferation Assays

CCK‐8 assay: Cell viability was detected using the CCK‐8 kit (Dojindo, Kyushu, Japan) according to the manufacturer's instructions. Briefly, transfected cells were seeded at 3000 cells/well in 96‐well plates. After 24 h, 10 μL of CCK‐8 solution was added to each well, and absorbance at 450 nm was measured using a microplate reader (BioTek, Winsooki, VT, USA) after 2 h of incubation at 37°C. IC_50_ values were determined using CCK‐8 assays after 48 h of cisplatin treatment (0–120 μM) and calculated by nonlinear regression using GraphPad Prism.

Colony formation assay: Cells were seeded at 300 cells/well in 6‐well plates, and the culture medium was replaced every 3 days. After 10 days of culture, cells were fixed with 4% paraformaldehyde for 15 min and stained with 0.1% crystal violet for 20 min. A digital camera was used to capture images of the stained colonies.

### 2.6 Apoptosis Assay

Flow cytometry analysis was performed using the Annexin V‐FITC/PI Apoptosis Detection Kit (BD Biosciences, Franklin Lakes, NJ, USA). Briefly, cells were collected and washed with pre‐chilled PBS. Annexin V‐FITC and PI staining solutions were added, and samples were incubated for 15 min at room temperature in the dark. Finally, the samples were analysed using a flow cytometer.

### 2.7 Immunoprecipitation (IP)

To detect the acetylation level of SMAD7, cells were lysed with lysis buffer. A portion of the lysate served as the ‘Input’ control. The remaining lysate was incubated with an anti‐SMAD7 antibody or an isotype IgG control overnight at 4°C with rotation. Protein A/G agarose beads were then added and incubated for an additional 4 h to capture antigen–antibody complexes. After washing the precipitates several times with pre‐chilled lysis buffer, loading buffer was added, and samples were boiled. Finally, western blotting was performed using an anti‐acetylated lysine antibody.

### 2.8 Chromatin Immunoprecipitation (ChIP)

ChIP was performed using the ChIP kit (Millipore, Burlington, MA, USA). Briefly, cells were crosslinked with 1% formaldehyde, and chromatin was fragmented by sonication into 200–500 bp fragments. The lysate was incubated with an anti‐H3K27ac antibody (ab4729, Abcam) or an anti‐IgG antibody (ab172730, Abcam) overnight at 4°C. Immune complexes were captured using Protein A/G magnetic beads. Quantitative analysis of specific regions of the SMAD7 promoter was performed by qPCR.

### 2.9 Dual‐Luciferase Reporter Assay

Based on the predicted HDAC2 binding site in the SMAD7 promoter fragment, wild‐type (SMAD7‐WT) and mutant (SMAD7‐MUT) reporter vectors were constructed. After 48 h of co‐transfection, cells transfected with the reporter plasmids were analysed for luciferase activity using the Dual‐Luciferase Reporter Assay System (Promega, Shanghai, China). Reporter gene activity was normalised to the ratio of firefly luciferase activity to Renilla luciferase activity.

### 2.10 Immunofluorescence (IF)

Cells were fixed with 4% paraformaldehyde, permeabilised with 0.1% Triton X‐100 for 30 min, and blocked with BSA for 15 min. Cells were then incubated with an anti‐β‐catenin (ab223075, Abcam) or an anti‐Ɣ‐H2AX (ab11174, Abcam) primary antibody overnight at 4°C, followed by incubation with the corresponding fluorescent secondary antibody (Cy3‐conjugated goat anti‐mouse antibody, ab97035, Abcam) for 1 h at room temperature in the dark. Cell nuclei were stained with DAPI. Finally, cells were observed and photographed under a confocal microscope.

### 2.11 Animal Experiment

All animal experimental procedures were approved by the Institutional Animal Ethics Committee of Zhejiang Center of Laboratory Animals. Female BALB/c nude mice aged 4–6 weeks (Zhejiang Center of Laboratory Animals) were randomly divided into four groups (*n* = 5): the control group, the cisplatin group, the sh‐HDAC2 group and sh‐HDAC2 + cisplatin group. For tumour inoculation, mice in all groups were subcutaneously injected with 5 × 10^6^ cells suspended in 100 μL of PBS/Matrigel (1:1) into the right flank. Specifically, the control group and cisplatin group received vector‐control A2780/DDP cells, whereas the sh‐HDAC2 group and sh‐HDAC2 + cisplatin group received stably transfected sh‐HDAC2 A2780/DDP cells. One week after inoculation, mice in the cisplatin group and sh‐HDAC2 + cisplatin group received intraperitoneal injections of cisplatin (3 mg/kg) every 3 days. Meanwhile, the control and sh‐HDAC2 groups received equal volumes of PBS. Tumour volume was measured every 3 days using the formula: Volume = (length × width/2) [2]. After 28 days of treatment, tumours were excised, imaged, and weighed.

### 2.12 Immunohistochemistry (IHC)

Mouse tumour tissue samples were fixed in 4% paraformaldehyde, embedded in paraffin, and sectioned. Sections were incubated with primary antibodies against Ki‐67 (1:200, ab16667, Abcam), HDAC2 (1:4000, ab219053, Abcam), N‐cadherin (1:200, ab98952, Abcam), MMP‐9 (1:1000, ab76003, Abcam) and E‐cadherin (1:500, ab40772, Abcam) overnight at 4°C. The following day, HRP‐conjugated secondary antibodies and a DAB chromogen kit were used for colour development. Cell nuclei were counterstained with haematoxylin. Finally, staining intensity was observed and evaluated under a light microscope.

### 2.13 Haematoxylin and Eosin (H&E) Staining

For histopathological evaluation, harvested tumour tissues were fixed in 10% neutral‐buffered formalin, dehydrated through graded alcohols, cleared in xylene and embedded in paraffin wax using standard protocols. Serial sections (4 μm thick) were cut using a microtome and mounted on glass slides. Tissue sections were stained with H&E according to standard procedures. Histopathological examination was performed under light microscopy, and representative photomicrographs were captured for morphological analysis.

### 2.14 Statistical Analysis

All experiments were independently repeated at least three times. Data are presented as the mean ± standard deviation. Statistical analysis was performed using GraphPad Prism 8.0 software. Comparisons between two groups were performed using Student's *t*‐test, while comparisons among multiple groups were performed using one‐way analysis of variance. A value of *p* < 0.05 was considered statistically significant.

## 3 Results

### 3.1 High HDAC2 Expression Promotes Cisplatin Resistance in OC Cells

**FIGURE 1:**
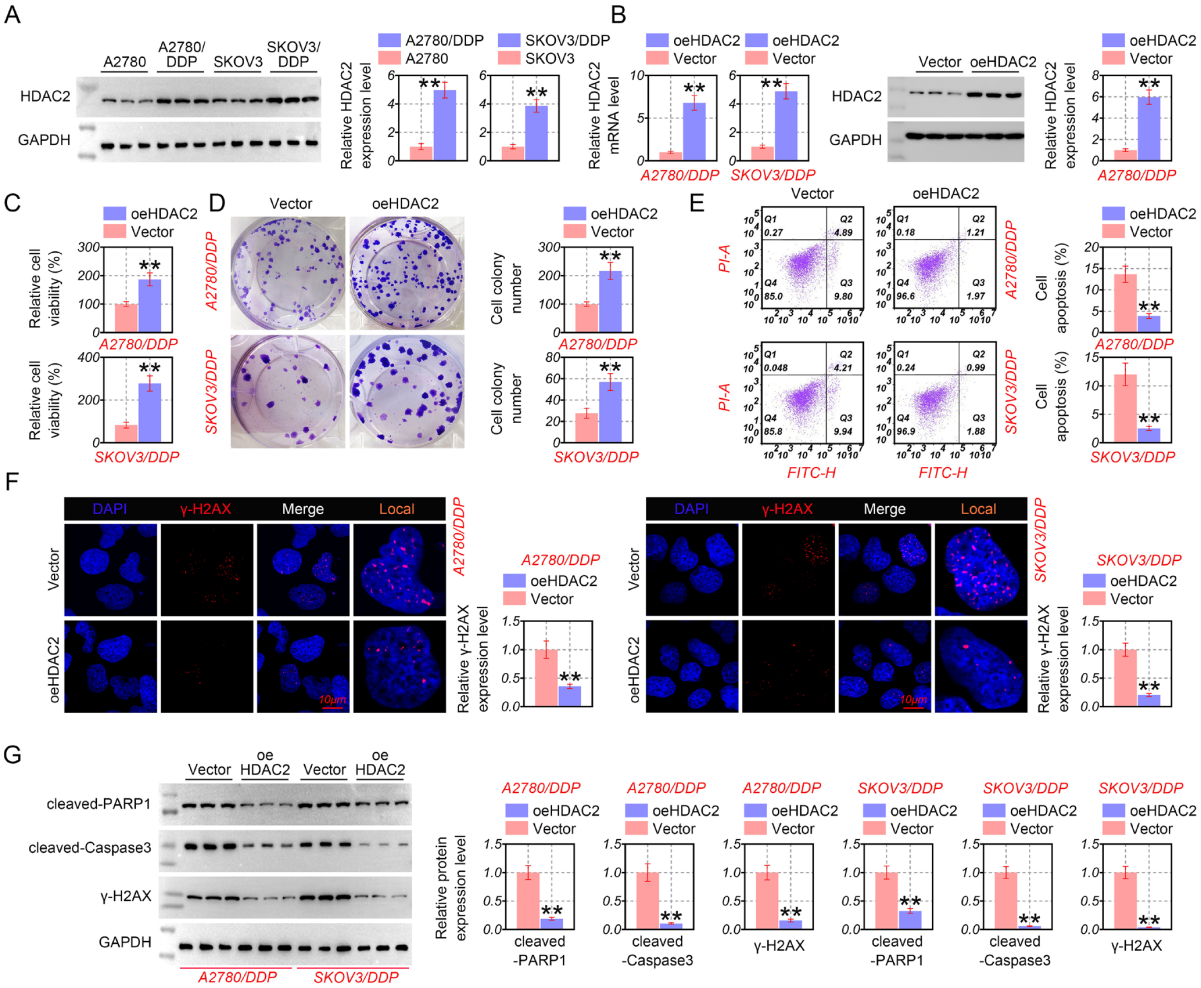
High HDAC2 expression promotes cisplatin resistance in OC cells. (A) HDAC2 expression levels in A2780, A2780/DDP, SKOV3 and SKOV3/DDP cells were measured by western blotting. **p < 0.01 versus A2780 or SKOV3. (B) A2780/DDP and SKOV3/DDP cells were divided into the Vector and oeHDAC2 groups. The mRNA and protein levels of HDAC2 were detected. (C) Cell viability was tested using the CCK‐8 assay. (D) Cell proliferative ability was measured using the colony formation assay. (E) Annexin V/PI staining assay was used to detect cell apoptosis. (F) The nuclear accumulation of Ɣ‐H2AX in OC cells was determined using the IF assay. Scale bar = 10 μm. (G) The protein levels of cleaved PARP1, cleaved caspase‐3 and Ɣ‐H2AX were detected. **p < 0.01 versus Vector. Data are presented as the mean ± SD from three independent experiments (*n* = 3 per group).

To investigate the association between HDAC2 and cisplatin resistance in OC, we first assessed HDAC2 expression in cisplatin‐resistant OC cell lines (A2780/DDP and SKOV3/DDP) and observed a significant upregulation (Figure 1A). Subsequently, we transfected HDAC2 overexpression vectors (oeHDAC2) into cisplatin‐resistant OC cells and validated the transfection efficiency via qRT‐PCR and western blotting. The results showed that both mRNA and protein levels of HDAC2 were markedly elevated, indicating successful construction of the overexpression model (Figure 1B). CCK‐8 assays demonstrated that HDAC2 overexpression significantly enhanced the viability of cisplatin‐resistant OC cells (Figure 1C), and this pro‐proliferative effect was further supported by a colony formation assay (Figure 1D). To directly quantify chemoresistance, we determined the IC_50_ values of cisplatin via CCK‐8 assays. In A2780/DDP cells, oeHDAC2 elevated the IC_50_ from 63.53 μM to 85.75 μM, while in SKOV3/DDP cells, it increased from 40.54 μM to 59.92 μM. Conversely, HDAC2 knockdown significantly reduced the IC_50_ values in both cell lines (Figure S1). Apoptosis analysis by Annexin V/PI staining revealed that HDAC2 overexpression inhibited cell apoptosis (Figure 1E). Moreover, the IF experiment showed that HDAC2 overexpression reduced nuclear accumulation of Ɣ‐H2AX (Figure 1F). Western blotting analysis further indicated that HDAC2 overexpression downregulated the expression of apoptosis‐related marker proteins (cleaved‐PARP1 and cleaved caspase‐3) as well as the DNA damage marker Ɣ‐H2AX (Figure 1G). These results suggest that elevated HDAC2 expression promotes cisplatin resistance in OC cells, potentially by attenuating DNA damage and suppressing apoptosis.

### 3.2 HDAC2 Knockdown Enhances Cisplatin Sensitivity in OC Cells

**FIGURE 2:**
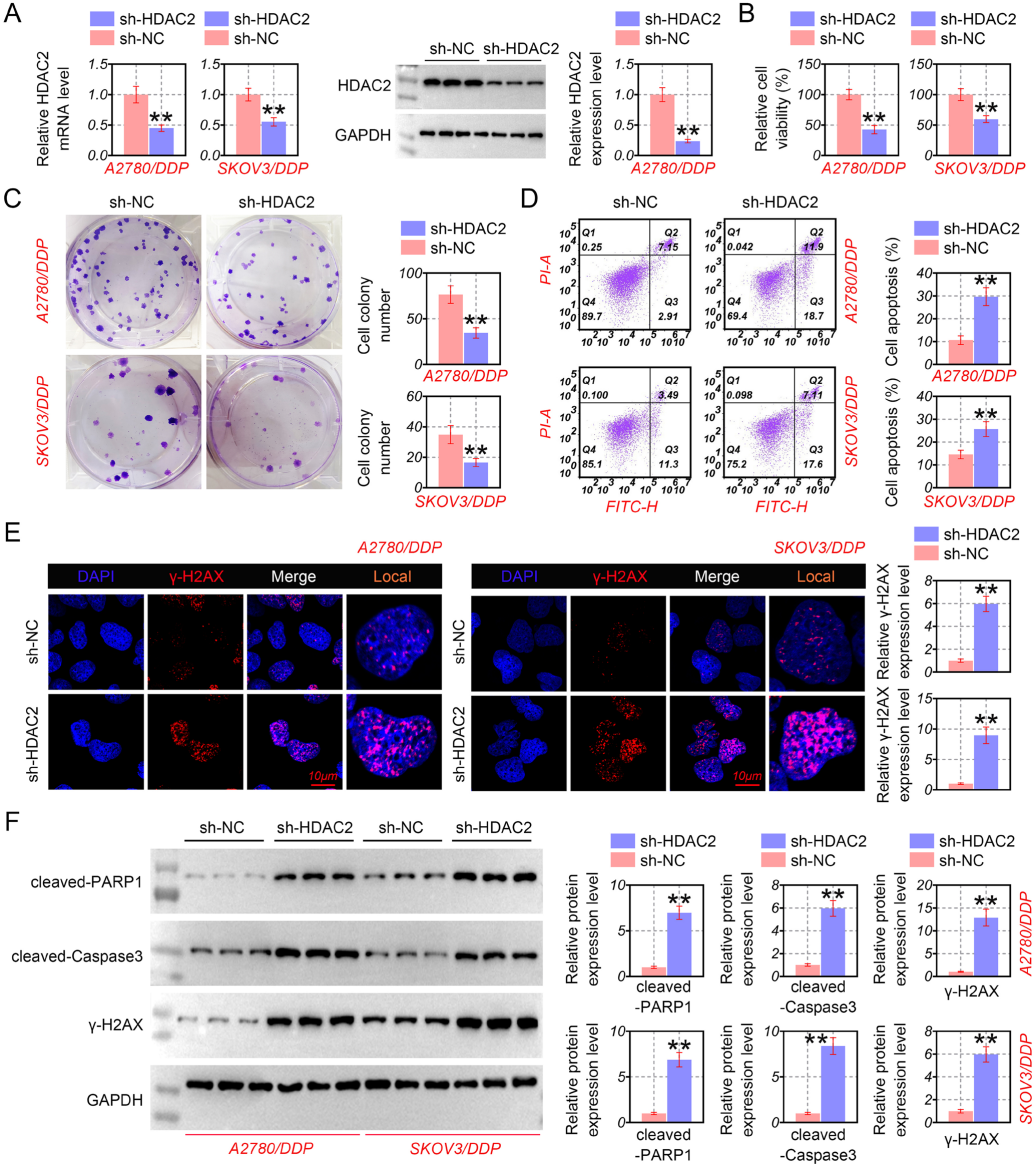
HDAC2 knockdown enhances cisplatin sensitivity in OC cells. A2780/DDP and SKOV3/DDP cells were divided into the sh‐NC and sh‐HDAC2 groups. (A) The mRNA and protein levels of HDAC2 were detected. (B) Cell viability. (C) The cell colony number. (D) The cell apoptosis. (E) Nuclear accumulation of Ɣ‐H2AX in OC cells. Scale bar = 10 μm. (F) The protein levels of cleaved PARP1, cleaved caspase‐3 and Ɣ‐H2AX. **p < 0.01 versus sh‐NC. Data are presented as the mean ± SD from three independent experiments (*n* = 3 per group).

We next explored whether HDAC2 knockdown could sensitise ovarian cancer cells to cisplatin. In cisplatin‐resistant OC cell lines (A2780/DDP and SKOV3/DDP), transfection with sh‐HDAC2 effectively suppressed HDAC2 expression at both the mRNA and protein levels, confirming efficient knockdown (Figure 2A). Functional assays demonstrated that HDAC2 knockdown significantly reduced cell viability (Figure 2B), inhibited proliferative capacity (Figure 2C), and promoted apoptosis (Figure 2D). Furthermore, HDAC2 knockdown enhanced nuclear accumulation of Ɣ‐H2AX (Figure 2E). Consistent with these observations, western blot analysis showed the upregulation of apoptosis‐related proteins (cleaved PARP1 and cleaved caspase‐3) and the DNA damage marker γ‐H2AX (Figure 2F). Collectively, these data indicate that HDAC2 knockdown enhances the sensitivity of OC cells to cisplatin by aggravating DNA damage and promoting apoptosis.

### 3.3 HDAC2 Catalyses H3K27 Deacetylation in the SMAD7 Promoter Region

**FIGURE 3:**
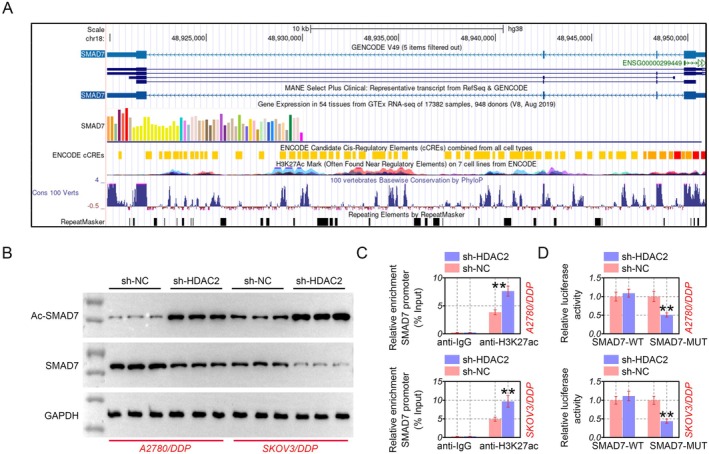
HDAC2 catalyses H3K27 deacetylation in the SMAD7 promoter region. (A) Schematic representation of the SMAD7 promoter region. (B) IP analysis of acetylated SMAD7 (Ac‐SMAD7) and total SMAD7 protein levels in cisplatin‐resistant OC cells (A2780/DDP and SKOV3/DDP) following HDAC2 knockdown. (C) ChIP assays quantifying the enrichment of H3K27ac in the SMAD7 promoter region after HDAC2 knockdown in A2780/DDP and SKOV3/DDP cells. (D) Dual‐luciferase reporter assay assessing the transcriptional activity of the SMAD7 promoter upon HDAC2 knockdown. **p < 0.01 versus sh‐NC. Data are presented as the mean ± SD from three independent experiments (*n* = 3 per group).

To investigate the potential transcriptional regulation of SMAD7, we examined the histone modification profiles within its promoter region. Analysis via the UCSC Genome Browser (https://genome.ucsc.edu/) revealed a notable enrichment of H3K27 acetylation (H3K27ac) within the SMAD7 promoter region (Figure 3A). To investigate the molecular basis, we performed co‐immunoprecipitation assays. As shown in (Figure 3B), HDAC2 knockdown markedly elevated SMAD7 acetylation (Ac‐SMAD7) but significantly diminished total SMAD7 protein abundance, indicating that HDAC2‐mediated deacetylation prevents SMAD7 from hyperacetylation‐induced degradation. This stabilisation mechanism also explains the seemingly paradoxical observations in the promoter assays: although HDAC2 knockdown increased H3K27ac enrichment at the SMAD7 promoter the promoter transcriptional activity was decreased. Collectively, these data suggest that the stability of the SMAD7 protein itself, rather than the local histone acetylation status, is the dominant factor governing SMAD7 functionality. Therefore, HDAC2 serves as a stabiliser of the SMAD7, ensuring sufficient protein levels to sustain downstream oncogenic signalling and chemoresistance.

### 3.4 The HDAC2/SMAD7 Axis Enhances Cisplatin Resistance in OC Cells by Promoting DNA Damage Repair

**FIGURE 4:**
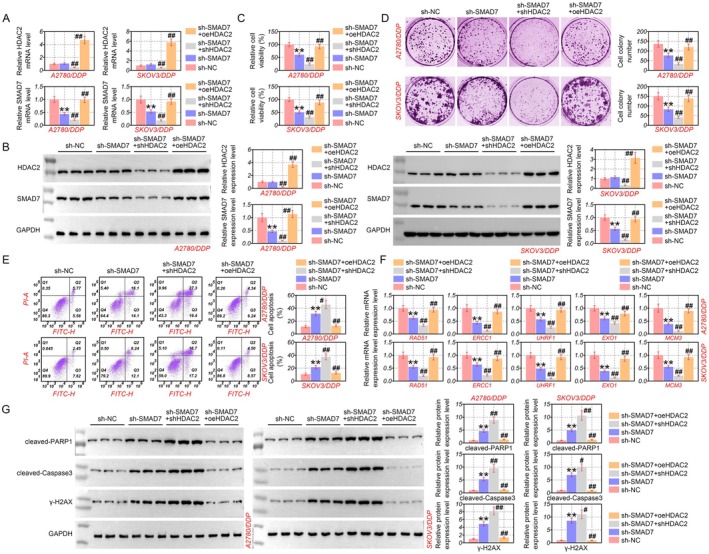
The HDAC2/SMAD7 axis enhances cisplatin resistance in OC cells by promoting DNA damage repair. A2780/DDP and SKOV3/DDP cells were divided into sh‐NC, sh‐SMAD7, sh‐SMAD7 + sh‐HDAC2, and sh‐SMAD7 + oeHDAC2 groups. (A) The mRNA levels of HDAC2 and SMAD7. (B) The protein levels of HDAC2 and SMAD7. (C) Cell viability. (D) Cell colony number. (E) Cell apoptosis. (F) The mRNA levels of RAD51, ERCC1, UHRF1, EXO1, and MCM3. (G) The protein levels of cleaved PARP1, cleaved caspase‐3 and Ɣ‐H2AX. **p < 0.01 versus sh‐NC; ##*p* < 0.001 versus sh‐SMAD7. Data are presented as the mean ± SD from three independent experiments (*n* = 3 per group).

To determine whether HDAC2 drives chemoresistance specifically through SMAD7, we performed rescue experiments. qRT‐PCR and western blot analyses confirmed that endogenous SMAD7 silencing (sh‐SMAD7) significantly reduced SMAD7 expression, an effect further exacerbated by concurrent knockdown of endogenous HDAC2 (sh‐HDAC2). Conversely, ectopic overexpression of HDAC2 (oeHDAC2) via lentiviral infection effectively restored SMAD7 protein levels in sh‐SMAD7 cells (Figure 4A,B). Functionally, SMAD7 knockdown markedly suppressed cell viability and proliferation, while promoting apoptosis (Figure 4C). Importantly, concurrent HDAC2 knockdown aggravated these tumour‐suppressive phenotypes (Figure 4D), whereas forced HDAC2 expression successfully reversed these effects (Figure 4C–E).

At the molecular level, SMAD7 knockdown downregulated the mRNA levels of DNA damage repair‐related genes (RAD51, ERCC1, UHRF1, EXO1, and MCM3). Similarly, this downregulation was further enhanced by HDAC2 knockdown but was markedly rescued by HDAC2 overexpression (Figure 4F). Western blotting also indicated that SMAD7 knockdown increased the protein levels of cleaved PARP1, cleaved caspase‐3, and γ‐H2AX. These increases were more pronounced with combined HDAC2 knockdown, whereas HDAC2 overexpression substantially counteracted their upregulation (Figure 4G). Together, these data demonstrate that the HDAC2/SMAD7 axis contributes to cisplatin resistance in OC cells by modulating the expression of DNA damage repair‐related genes.

### 3.5 The HDAC2/SMAD7 Axis Regulates the Wnt/β‐Catenin Signalling Pathway

**FIGURE 5:**
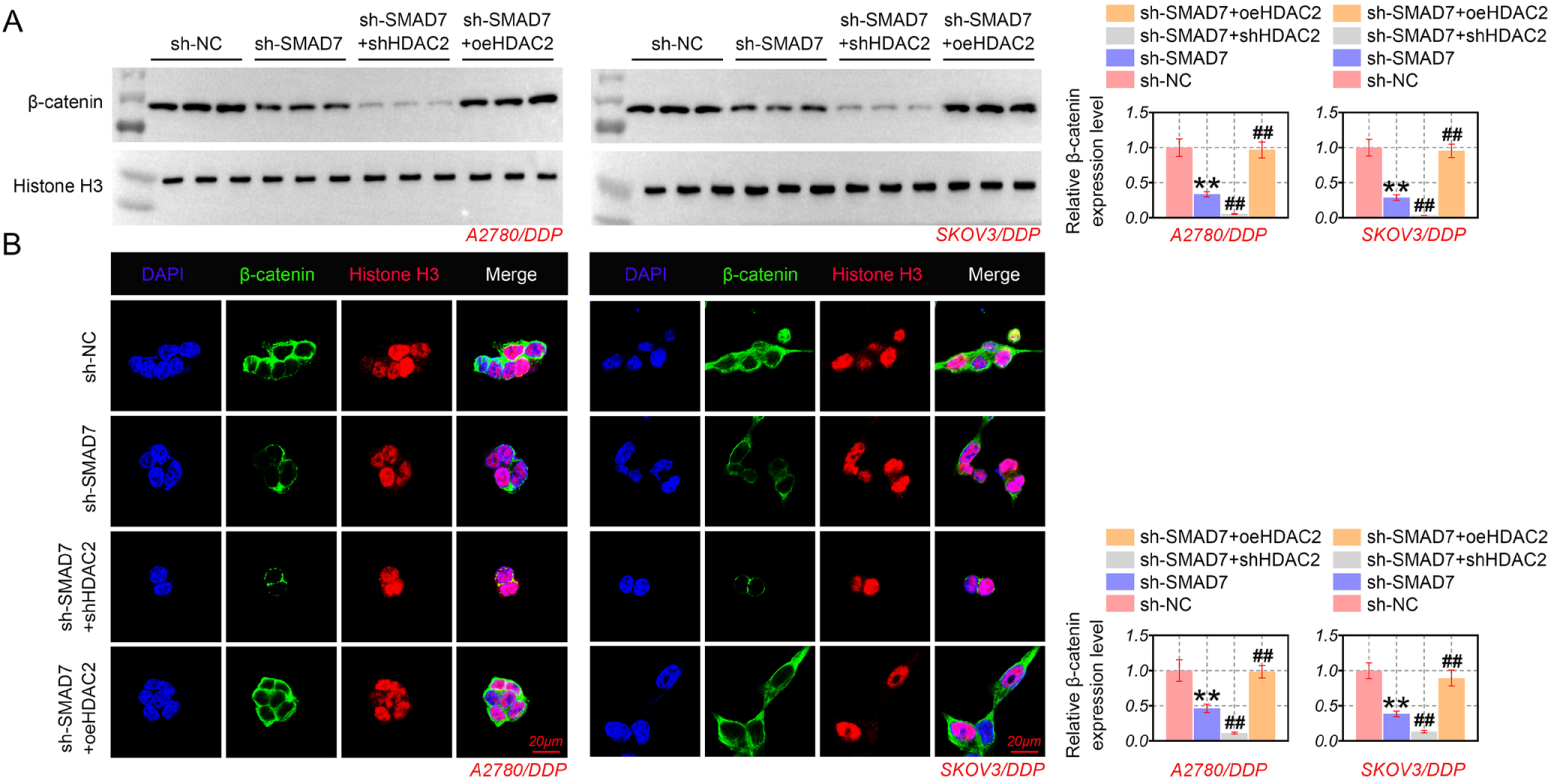
The HDAC2/SMAD7 axis regulates the Wnt/β‐catenin signalling pathway. A2780/DDP and SKOV3/DDP cells were divided into sh‐NC, sh‐SMAD7, sh‐SMAD7 + sh‐HDAC2, and sh‐SMAD7 + oeHDAC2 groups. (A) Protein level of nuclear β‐catenin was detected using western blotting. (B) IF staining was used to observe the localisation of β‐catenin. Scale bar = 20 μm. **p < 0.01 versus sh‐NC; ##*p* < 0.001 versus sh‐SMAD7.

To further investigate the downstream mechanisms of the HDAC2/SMAD7 axis in cisplatin resistance, we examined its effect on the Wnt/β‐catenin pathway. Experimental results revealed that nuclear β‐catenin protein levels were substantially reduced upon SMAD7 knockdown. This reduction was further enhanced by combined HDAC2 knockdown, whereas HDAC2 overexpression effectively restored nuclear β‐catenin expression (Figure 5A). The alterations in nuclear β‐catenin were further confirmed by IF analysis (Figure 5B). These findings demonstrate that the HDAC2/SMAD7 axis functions as an upstream regulator of the Wnt/β‐catenin signalling pathway in cisplatin‐resistant OC cells.

### 3.6 HDAC2 Knockdown Enhances Cisplatin Sensitivity in OC in Vivo

**FIGURE 6:**
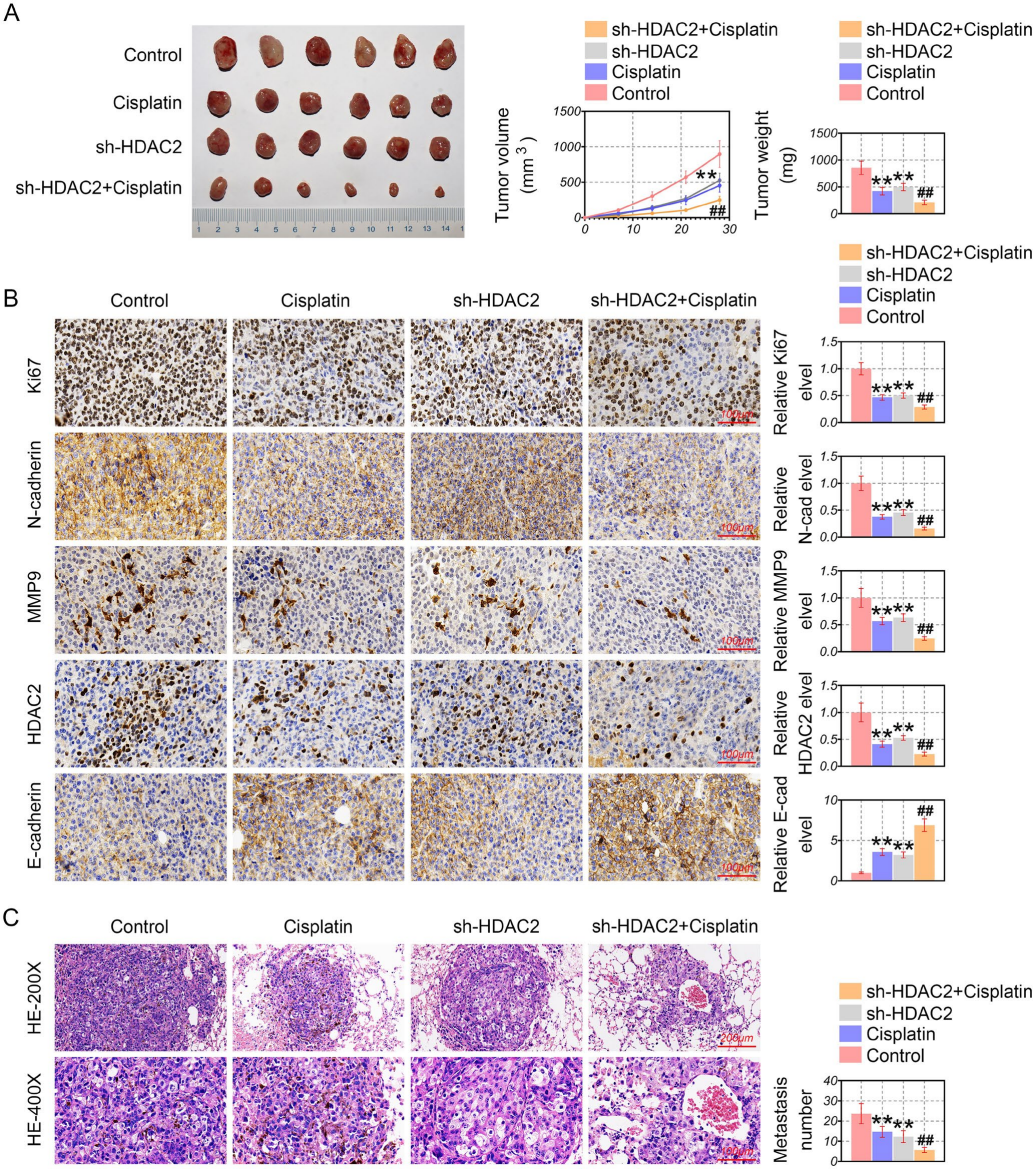
HDAC2 knockdown enhances cisplatin sensitivity in OC in vivo. Mice were divided into Control, Cisplatin, sh‐HDAC2, and sh‐HDAC2 + Cisplatin groups. (A) Tumour volume and weight. (B) Representative IHC staining images showing the expression of Ki‐67, HDAC2, E‐cadherin, N‐cadherin and MMP‐9 in tumour tissues from the different treatment groups. Scale bar = 100 μm. (C) H&E staining of tumour tissues. Scale bar = 200 μm (upper). Scale bar = 100 μm (down). **p < 0.01 versus Control; ##*p* < 0.001 versus sh‐SMAD7 + Cisplatin. Data are presented as the mean ± SD from three independent experiments (*n* = 6 per group).

To validate the effects of HDAC2 on cisplatin resistance in vivo, we established a mouse xenograft model using OC cells (A2780/DDP). The results showed that both cisplatin treatment and sh‐HDAC2 alone significantly reduced tumour volume and weight compared with those of the control group. Notably, the combination of sh‐HDAC2 and cisplatin resulted in the most substantial suppression of tumour growth (Figure 6A). IHC analysis indicated that either cisplatin or sh‐HDAC2 treatment downregulated the expression of Ki‐67, HDAC2, N‐cadherin, and MMP‐9, while upregulating E‐cadherin. These regulatory effects were more pronounced in the combination treatment group (Figure 6B). Furthermore, H&E staining revealed that both individual treatments inhibited OC metastasis, with the strongest inhibitory effect observed in the combination group (Figure 6C). In summary, HDAC2 knockdown enhances the sensitivity of OC to cisplatin in vivo and exerts a synergistic anti‐tumour effect when combined with cisplatin.

## 4 Discussion

Chemotherapy resistance, particularly to platinum‐based agents such as cisplatin, remains a major obstacle in OC treatment [4]. This study focused on HDAC2 and systematically elucidated how HDAC2‐mediated post‐translational regulation drives cisplatin resistance in OC. Our findings provide novel mechanistic insights and suggest potential therapeutic strategies for overcoming chemoresistance.

This study first established a positive correlation between HDAC2 expression levels and cisplatin resistance in OC and confirmed, through gain‐ and loss‐of‐function experiments that HDAC2 is a direct driver of the resistant phenotype. This finding is consistent with other cancers research. For instance, in non‐small cell lung cancer, HDAC2 expression correlates positively with cisplatin resistance, and inhibiting HDAC2 enhances cisplatin‐induced apoptosis [12]. Studies in gastric and hepatocellular carcinoma have demonstrated that the combination of HDAC inhibitors with cisplatin significantly suppresses the survival and proliferation of resistant cells [13, 14]. Our research extends these findings to OC, establishing that high HDAC2 expression is not merely associated with cisplatin resistance in OC, but rather an important pathogenic factor.

Regarding mechanistic exploration, to elucidate the molecular mechanisms by which HDAC2 mediates resistance, we directed our research focus toward downstream targets and discovered that HDAC2 primarily regulates SMAD7 at the post‐translational level. As a histone deacetylase, the core function of HDAC2 is to catalyse histone deacetylation, typically leading to chromatin compaction and transcriptional repression [6]. H3K27ac is a canonical active transcription mark, usually enriched at the promoters and enhancers of active genes [15]. In this study, we observed prominent H3K27 acetylation enrichment in the SMAD7 promoter region; however, HDAC2 knockdown unexpectedly reduced, rather than increased SMAD7 promoter transcriptional activity and total SMAD7 protein levels, suggesting that H3K27ac modulation is not the dominant mechanism in this context. Instead, our results indicate that HDAC2 directly deacetylates and stabilises the SMAD7 protein, preventing its hyperacetylation‐induced degradation. This finding is of considerable significance because it links HDAC2‐mediated protein stability with SMAD7, a key negative regulator of the TGF‐β signalling pathway [16]. Previous studies have largely concentrated on the role of HDACs in regulating classical oncogenes or tumour suppressors (such as p21) [17, 18], whereas we identified a novel mechanism whereby HDAC2 protects SMAD7 from degradation, providing a new perspective for understanding resistance.

To elucidate the biological function of the HDAC2/SMAD7 axis, we performed a series of related experiments and discovered a novel function of the HDAC2/SMAD7 axis in regulating DNA damage repair. Cisplatin primarily kills tumour cells by inducing DNA interstrand crosslinks; therefore, efficient DNA damage repair capacity is one of the core mechanisms by which cancer cells develop resistance [19]. We found that SMAD7 knockdown significantly suppressed the expression of DNA damage repair‐related genes and this effect could be reversed by HDAC2 overexpression. This indicates that the maintenance of SMAD7 expression (via HDAC2‐mediated stabilisation) is a necessary component for HDAC2 to promote DNA damage repair and the resistant phenotype. Additionally, we discovered that the Wnt/β‐catenin signalling pathway is a downstream effector of the HDAC2/SMAD7 axis. Activation of the Wnt/β‐catenin signalling pathway plays an important role in OC development and progression, which can be estimated by nuclear β‐catenin protein levels [20, 21]. This study found that the HDAC2/SMAD7 axis regulates β‐catenin nuclear localisation. This provides a novel explanation for HDAC2‐mediated cisplatin resistance: HDAC2 indirectly activates the Wnt/β‐catenin pathway by stabilising SMAD7 protein, thereby enhancing the survival capacity and drug resistance of OC cells.

Finally, our in vivo animal experiments strongly supported the conclusions from in vitro studies. In nude mouse xenograft models, HDAC2 knockdown significantly inhibited tumour growth and metastasis, generating synergistic effects with cisplatin treatment. IHC analysis further demonstrated that combined treatment most effectively reduced the expression of the proliferation marker Ki‐67 and EMT‐related proteins (N‐cadherin, MMP‐9) while upregulating the epithelial marker E‐cadherin. This not only validated the feasibility of HDAC2 as a therapeutic target but also suggested that its regulation of resistance may involve multiple mechanisms, including inhibition of proliferation and reversal of EMT. These in vivo data provide solid preclinical evidence for HDAC2 as a potential therapeutic target for reversing cisplatin resistance in OC.

However, this study has certain limitations. For instance, although this study discovered that the HDAC2/SMAD7 axis affects the Wnt/β‐catenin pathway activity, how SMAD7 crosstalks with the Wnt pathway and the specific molecular bridges involved have not been elucidated. All functional validations were completed in cell lines and mouse models, and the clinical relevance, and correlation of this axis with OC patient tissues have not yet been investigated. Future research will be dedicated to addressing these limitations, providing more robust and comprehensive theoretical foundation for HDAC2 as a target for reversing cisplatin resistance in OC.

## 5 Conclusion

In summary, this study systematically revealed the molecular mechanisms by which HDAC2 mediates cisplatin resistance in OC. Our research demonstrates that HDAC2 functions as a critical stabiliser of the SMAD7 protein by directly mediating its deacetylation, thereby preventing hyperacetylation‐induced degradation. The maintenance of SMAD7 protein levels subsequently drives cisplatin resistance in OC by enhancing DNA damage repair capacity and potentially activating the Wnt/β‐catenin signalling pathway. These findings not only deepen our understanding of the mechanisms underlying chemotherapy resistance in OC but also provide novel strategies and targets for overcoming cisplatin resistance.

## Author Contributions

**Yingying He:** investigation, conceptualization, funding acquisition, writing – original draft, writing – review and editing, validation, methodology, visualization, project administration, formal analysis, software, data curation, supervision, resources. **Kang Zheng:** investigation, methodology, validation, visualization, project administration, formal analysis, software, data curation, supervision, resources. **Kening Zhou:** conceptualization, writing – original draft, writing – review and editing. **Xiaomin Xu:** investigation, visualization, validation, methodology, software, formal analysis, project administration, resources, supervision, data curation. **Meiling Wu:** investigation, visualization, validation, methodology, software, formal analysis, project administration, resources, supervision, data curation.

## Funding

This work was supported by Quzhou City Competitive Project (Grant no. 2025K062).

## Ethics Statement

Ethical approval was obtained from the Ethics Committee of Zhejiang Center of Laboratory Animals.

## Consent

Written informed consent was obtained from a legally authorised representative(s) for anonymised patient information to be published in this article.

## Conflicts of Interest

The authors declare no conflicts of interest.

## Supporting information


**Figure S1:** HDAC2 modulates the sensitivity of OC cells to cisplatin. The IC_50_ values of cisplatin were determined by CCK‐8 assays in A2780/DDP and SKOV3/DDP cells with HDAC2 overexpression (oeHDAC2) or knockdown (sh‐HDAC2). Cells were exposed to gradient concentrations of cisplatin (0–120 μM) for 48 h. **p < 0.01 vs. Vector; ##*p* < 0.01 vs. sh‐NC. Data are presented as mean ± SD from three independent experiments (*n* = 3 per group).

## Data Availability

The data that support the findings of this study are available from the corresponding author upon reasonable request.
